# The many roads to psychosis: recent advances in understanding risk and mechanisms

**DOI:** 10.12688/f1000research.16574.1

**Published:** 2018-12-03

**Authors:** Carrie E. Bearden, Jennifer K. Forsyth

**Affiliations:** 1 Department of Psychiatry and Behavioral Sciences, Semel Institute for Neuroscience and Human Behavior, University of California, Los Angeles, CA, USA; 2 Department of Psychology, University of California, Los Angeles, CA, USA; 3 Center for Neurobehavioral Genetics, University of California, Los Angeles, CA, USA

**Keywords:** neurodevelopment, psychosis, synaptic function, clinical high risk

## Abstract

Schizophrenia is a chronic and severe mental illness which frequently leads to substantial lifelong disability. The past five years have seen major progress in our understanding of the complex genetic architecture of this disorder. Two major barriers to understanding the core biological processes that underlie schizophrenia and developing better interventions are (1) the absence of etiologically defined biomarkers and (2) the clinical and genetic heterogeneity of the disorder. Here, we review recent advances that have led to changes in our understanding of risk factors and mechanisms involved in the development of schizophrenia. In particular, mechanistic and clinically oriented approaches have now converged on a focus on disruptions in early neurodevelopment and synaptic plasticity as being critical for both understanding trajectories and intervening to change them. Translating these new findings into treatments that substantively change the lives of patients is the next major challenge for the field.

## Introduction

Schizophrenia is a severe and highly disabling chronic mental illness involving disordered thought and perception. Its characteristic onset in late adolescence/early adulthood is often associated with a marked, lifelong impact on social and role functioning. At the same time, there is a wealth of evidence from epidemiologic birth cohort and retrospective studies that at least a proportion of individuals who ultimately develop schizophrenia have more subtle deficits in cognitive, social-affective, motor, and language development in early childhood, long before the onset of overt illness. This would suggest that (1) onset of overt symptoms is a “late stage” of illness, providing a window of opportunity to intervene *if* those at risk can be accurately identified, and (2) schizophrenia, despite its later age at onset, may be considered a disorder of disrupted neurodevelopment.

In the past decade, significant advances in our understanding of the genetic architecture of schizophrenia have confirmed intuitions that convergent biological pathways across disparate genetic risk factors implicate genes involved in synaptic plasticity and neuronal development. Yet, despite this impressive body of new knowledge regarding risk genes for schizophrenia, we still have a very incomplete understanding of mechanism. Two major obstacles to elucidating the core biological processes that underlie schizophrenia are (1) the absence of objective biomarkers and (2) the clinical and genetic heterogeneity of the disorder ^1^. These also impede our ability to develop better and more mechanistically informed interventions.

Here, we will discuss the major discoveries of the past few years and relevant clinical advances. The emerging framework is one of disrupted neurodevelopment involving both early, subtle anomalies and progressive maturational disturbances, namely accelerated gray matter loss in regions critical for higher-order cognition ^2^. Studies of youth at clinical high risk (CHR) for developing psychosis have greatly informed our understanding of risk factors, the importance of early intervention, and potential mechanisms proximal to illness onset, but there are also challenges. In particular, potential bias in recruitment strategies and late identification of at-risk individuals pose potential limitations ^3^. As such, we need alternative approaches to identify those at highest risk earlier in development. As we will discuss in detail below, examining convergence of clinically defined risk with rare genetic variants that are highly penetrant for illness can better advance mechanistic understanding of psychosis risk. However, key challenges for the field remain, namely how we translate scientific advances into real improvements in clinical care and disease prognosis.

## The genetic architecture of schizophrenia

For many years, it was known, based on consistent evidence from twin and family studies, that schizophrenia is highly heritable ^4– 7^. As such, there was a widely held view that understanding the genetics of schizophrenia might provide a window into the disease biology. Given the prevalence of schizophrenia (about 1% in the general population) and its genetic inheritance pattern, investigators proposed a “common-disease, common-allele” model ^8^, in which illness results from the cumulative effect of multiple common alleles. Yet, even in studies of thousands of individuals, no genome-wide significant risk variants had been identified. However, karyotypic abnormalities had been detected in affected individuals or families ^9^, suggesting the possibility of some major mutational causes of schizophrenia. The advent of microarray-based methodology recently enabled the detection of much smaller structural genetic events. In 2008, our understanding of the genetic architecture changed dramatically with the findings of Walsh *et al.* ^10^, who reported that some mutations predisposing to schizophrenia are highly penetrant (odds ratios range from 2 to 30), individually rare, and evolutionarily recent. In rapid succession, several larger consortia studies confirmed and replicated these findings, revealing that submicroscopic copy number variations (CNVs)—including recurrent deletions at 1q21.11, 15q11.3, 22q11.2, and the neurexin 1 locus at 2p16.3—are associated with greatly increased risk for psychosis as well as other developmental neuropsychiatric disorders and intellectual disability ^11– 13^. These CNVs occur disproportionately in loci containing genes involved in synaptic function and neurodevelopment, including neuregulin and glutamate pathways, as well as the post-synaptic density, including the activity-regulated cytoskeleton (ARC) protein complex, which localizes to NMDAR (N-methyl-D-aspartate receptor)-activated synapses and plays a role in plasticity-induced cytoskeleton changes ^14^. Yet, collectively, these risk loci are carried by less than 2.5% of patients and thus do not explain the majority of cases of schizophrenia. In 2014, in a genome-wide association study (GWAS) of about 36,000 patients and over 100,000 controls, the Psychiatric Genomics Consortium ^15^ reported the very first set of genome-wide significant findings for *common* schizophrenia risk variants. This study implicated over 100 loci but all with individually small effect sizes. These risk variants were enriched for brain-expressed genes and for genes relevant to synaptic plasticity and immune function, particularly within the major histocompatibility complex (MHC) region. Subsequently, Sekar *et al.* ^16^ demonstrated an association between risk for schizophrenia and genetic variation that alters expression of particular forms of complement component 4 (C4), part of the innate immune system. Importantly, the authors linked these genetic findings to schizophrenia pathophysiology for the first time, as they showed that loss of C4 resulted in reduced post-natal synaptic pruning in a mouse model. Given that *increased* expression of CA4a is associated with greater risk of schizophrenia, these findings support the long-standing hypothesis that disrupted synaptic refinement (that is, perhaps overly aggressive synaptic pruning) in adolescence plays a key role in disease etiology ^17^.

This rapid progress has continued with advances in data sharing, meta-analytic methods, and hence the publication of ever-larger GWASs and exome sequencing studies. Across the allelic frequency spectrum, this recent work consistently implicates biological pathways involved in synaptic functions; indeed, Genovese *et al.* ^18^ recently found that potentially synaptic genes appear to explain more than 70% of the exome enrichment in damaging ultra-rare variants contributing to schizophrenia. Synaptic plasticity, while critical for learning and memory across the life span, is also critically involved in development, as it plays a key role in organizing neurons into finely tuned circuits required for a mature brain ^2^.

The individually small but cumulative effects of multiple risk genes found in GWASs led to the development and increasing popularity of leveraging polygenic risk scores (PRS) for schizophrenia (i.e. calculating the total number of independent risk alleles, weighted by their effect sizes on disease, carried by an individual) ^19, 20^. The schizophrenia PRS has become an important potential “summary measure” for use in assessing risk and possibly future stratification in clinical trials ^21^.

**Figure 1.**
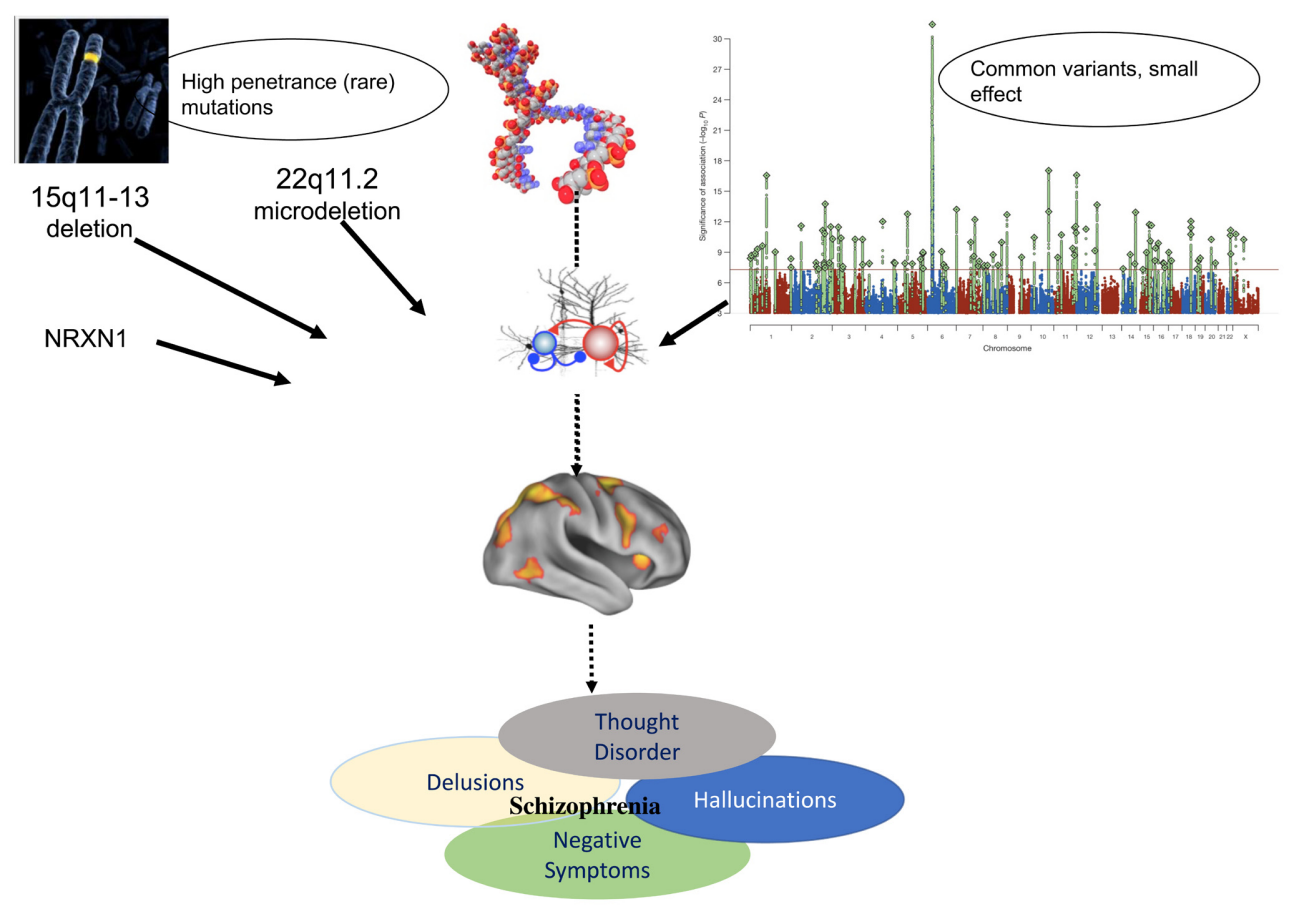
Convergence of rare and common genetic variation on disrupted synaptic function and neurodevelopmental processes. Convergence of rare and common genetic variation on disrupted synaptic function and neurodevelopmental processes. Top right-hand panel reprinted by permission from: Springer Nature. *Nature*. Biological insights from 108 schizophrenia-associated genetic loci. Schizophrenia Working Group of the Psychiatric Genomics Consortium, Stephan Ripke, Benjamin M. Neale, Aiden Corvin, James T. R. Walters *et al.*, 2014.

Nevertheless, given that no single common variant has an individually large effect, rare damaging variants and recurrent CNVs like the 22q11.2 microdeletion that are highly penetrant for psychosis may offer a clearer path toward mechanistic understanding ( Figure 1). Moreover, as such mutations can be detected very early in development (even *in utero*), they offer a unique opportunity for prospective study of neurodevelopmental influences on the evolution of psychosis, long before disease-related processes begin to unfold. As sample sizes increase, additional rare variants are likely to be discovered via sequencing methodologies; these genes are likely to cluster in similar pathways as the known risk variants but may also suggest additional biological processes.

Recently, Tansey *et al.* ^22^ identified a way in which these differing genetic mechanisms may converge: specifically, relative to controls, individuals with schizophrenia with known pathogenic CNVs also have an excess burden of common risk alleles. This finding supports a polygenic threshold model of schizophrenia, meaning that multiple risk variants may converge to reach a “risk threshold”, in contrast to an extreme heterogeneity model in which carriers of certain high-penetrance mutations form distinct subgroups. Importantly, these results support the notion that studies of rare variation may reveal pathophysiological mechanisms relevant to the broader population.

Overall, these large-scale genomic studies suggest that (1) schizophrenia is highly polygenic, involving hundreds to thousands of genes, and both common and rare variants confer risk; (2) schizophrenia risk variants are enriched for biological pathways that include synaptic development and plasticity as well as glutamatergic signaling ^23^; and (3) these risk variants partially overlap with those implicated in other developmental disorders, notably autism spectrum disorder and intellectual/developmental disability. Collectively, these recent findings support an emerging framework of lifelong biological vulnerability, which sets the stage for events during adolescence—including abnormal pruning and increased cortical dysconnectivity—that lead to neural disruption and clinical symptoms.

Nevertheless, there is considerable variability in course and outcome. Although psychosis onset is most common in late adolescence or early adulthood, some patients experience their first psychotic episode as early as childhood or after 40 years of age. Similarly, subsets of patients show a rapid onset of cognitive and functional decline in adolescence or early adulthood whereas others show poor cognitive function much earlier in life. This clinical variability may be related to variability in genetic profiles and heterogeneous molecular pathways: in particular, one hypothesis is that a relatively greater “rare variant” contribution may be present in patients with both schizophrenia and cognitive impairment and may characterize those patients with poorer premorbid function; this possibility is partially supported by Singh *et al.* ^24^, who recently found that the burden of rare, damaging variants is greater in patients with both schizophrenia and intellectual disability. However, this excess of rare, loss-of-function intolerant variants relative to controls is also seen in patients with schizophrenia who do not have intellectual disability, suggesting a “risk continuum”.

## Early identification and intervention: psychosis as a late stage

In parallel to these large-scale genetics discoveries, clinical advances over the past decade have focused on earlier identification and intervention, based on increasing evidence that reducing the duration of untreated psychosis results in better outcome, in terms of treatment response and both short- and longer-term role functioning ^25– 27^. Indeed, beginning in the mid-1990s, diagnostic criteria aimed at identifying individuals at high risk for imminent development of psychosis were established and applied in a number of clinical research studies ^28, 29^. This work is founded on the well-established “clinical staging” principle in medicine, which has shown that, for a number of common diseases, the invasiveness of the intervention scales with its timing, such that more benign (and low-cost) treatments are likely to be effective in the very early, “pre-onset” stages. Beginning with the implementation of early detection programs in Australia ^30, 31^, this concept rapidly expanded to Europe and the US. Overall, rates of conversion to overt psychosis in this identified CHR group range from 20 to 40% during the first three years after ascertainment ^28, 32^. Among those who do not develop a psychotic disorder, outcomes are highly variable; about one third continue to display significant subthreshold symptoms and functional impairment, and one third show symptomatic remission ^33^. Lower levels of negative mood and anxiety symptoms were associated with greater likelihood of both symptomatic and functional recovery.

A prospective multisite study, the North American Prodromal Longitudinal (NAPLS) consortium ^34^, has been prospectively following CHR youth with repeated clinical, cognitive, and imaging and blood biomarker assays and aims to improve our ability to predict conversion to psychosis so that preventive interventions can be offered to those who most need them. Recently, the NAPLS consortium found a set of replicable clinical and cognitive risk factors that predicted risk of conversion to psychosis on an individual basis: specifically, higher levels of unusual thought content and suspiciousness, poorer verbal learning and memory and information-processing speed, decline in social functioning, and younger age at baseline each contributed to individual risk for psychosis ^35^. The multivariate model achieved an overall accuracy that was comparable to that of individual prediction models for cardiovascular disease and cancer recurrence and was validated in an independent external data set ^36^. Based on these results, a web-based risk prediction tool was made available for predicting individual risk of conversion to psychosis in clinically ascertained cohorts.

Additionally, an important potential imaging biomarker for risk prediction emerged from this multisite project, which found that those who converted to psychosis over the follow-up period showed a significantly greater rate of gray matter loss than those who were symptomatic at baseline but did not convert ^37^. Gray matter loss was correlated with pro-inflammatory cytokines in plasma, suggesting a possible neuro-inflammatory mechanism. The discovery that variation in C4—part of the innate immune system—is causally involved in synaptic pruning ^16^ suggests a possible mechanistic basis for this finding. Future studies including the addition of imaging and potentially genetic biomarkers (for example, high polygenic risk for schizophrenia) to the calculator model are warranted to determine whether such factors account for additional variance.

Despite these advances, the proposal to include “psychosis risk syndrome” as a diagnostic category in the fifth edition of the *Diagnostic and Statistical Manual of Mental Disorders* remained controversial and ultimately was not included. Instead, “attenuated psychosis syndrome” was included as a “condition for further study”. Some of the reservations regarding its inclusion are that (1) specialized CHR services detect a only small minority of those who will ultimately develop psychosis, suggesting problems with ascertainment which may bias our understanding of mechanistic pathways to psychosis ^3^, (2) a substantial portion of individuals identified as CHR convert to psychosis shortly after ascertainment, and thus may be too advanced in the illness process to alter the trajectory, and (3) the majority of individuals who meet CHR criteria do not go on to develop a full-blown psychotic disorder ^28^; thus, the “psychosis risk syndrome” label may imply a greater level of risk than is currently warranted by the data.

There is now a consensus that “attenuated psychosis syndrome” is a clinically useful concept, as it identifies help-seeking individuals in need of intervention, who have elevated risk of developing a psychosis spectrum condition in the years following ascertainment ^38^. Importantly, the slow “ramp-up” to onset of the initial psychotic episode represents both a window of vulnerability and an opportunity to intervene. Findings of altered trajectories of brain development, proximal to and even prior to illness onset, have highlighted the possible role of progressive neuromaturational disturbances in the etiology of illness ^37^. The field has moved toward a clinical staging model, in which the onset of overt psychosis is considered a “late stage” of the illness ^1, 30^. Current guidelines recommend psychosocially focused interventions (cognitive behavioral therapy and family intervention) ^39, 40^ as a “first line” of treatment in this early stage of illness, and antipsychotic medication is recommended only in cases of severe and progressive symptomatology ^41^. Currently, there is no evidence that any particular intervention in CHR is superior to the others; however, there are few long-term outcome trials and results are inconsistent. More importantly, there is not yet a known mechanism of action for treatments that do show efficacy ^42^. Clearly, more work is needed to develop effective and efficient evidence-based prevention strategies that are based on individual prognosis and risk factors.

## The broader psychosis spectrum

In addition to increased focus on clinical ascertainment of at-risk individuals, there has been a conceptual “broadening” of the psychosis spectrum. Late adolescence to early adulthood represents a period of elevated risk for schizophrenia and related psychotic spectrum (PS) conditions ^43, 44^. In addition to being associated with the onset of clinically diagnosed psychotic illness, adolescence is associated with a peak in the emergence of subclinical psychotic-like experiences ^45, 46^. Unlike the break with external reality that defines psychosis, these experiences reflect mild to moderate deviations of beliefs and experiences from reality.

Recent evidence from population-based cohorts indicates that non-treatment-seeking youth experiencing PS symptoms exhibit changes in neural structure and function as well as alterations in social and cognitive processing that are qualitatively similar to differences observed in schizophrenia but of lesser magnitude ^47– 49^. In particular, PS youth show lower predicted cognitive age and greater developmental lag compared with both typically developing youth and those with other psychiatric symptoms ^50^, suggesting both possible etiologic overlap with syndromal psychosis and some degree of specificity. Furthermore, alterations in structural ^51^ and functional ^47, 52^ brain connectivity in PS youth seem to parallel those observed in established illness. New data from a large-scale multisite study of almost 4000 prepubertal youth indicate that more severe self-reported childhood psychotic-like experiences were associated with neurocognitive deficits, motor and speech developmental milestone delays, and family history of psychosis. These findings suggest that these symptoms, even in young children, are associated with many of the same risk factors associated with psychotic symptoms in older individuals, further supporting the dimensionality of psychosis across the life span ^53^.

## Conclusions

The past few years have seen remarkable discoveries regarding the genetic basis of schizophrenia. Yet, thus far, these advances have not led to new, mechanistically defined treatments for this devastating illness. Relatedly, very few biomarkers have been successfully implemented in clinical practice ^54^. How do we move forward to close the translational gap? Clearly, the complexity of schizophrenia’s genetic architecture, involving risk genes ranging from common alleles of small effect to rare alleles of large effect, presents major challenges. One key challenge will be identifying biological pathways that may converge across this “risk continuum”. Recent findings suggest that increased *C4A* expression in individuals with schizophrenia may result in overly aggressive synaptic pruning, leading to excessive gray matter loss proximal to disease onset. Although this intriguing possibility is unlikely to explain all cases of schizophrenia (perhaps not even a substantial percentage) and modification of synaptic pruning may pose challenges as a therapeutic target, this may be considered one of the first real inroads into the pathobiology of schizophrenia. In parallel, early intervention for those at CHR has improved our understanding of risk factors prior to overt illness onset and supports the efficacy of psychosocial interventions during this vulnerable period. As such, these mechanistic and clinically oriented approaches have now converged on a focus on early development as being critical for both understanding trajectories and intervening to change them.

Given the rapidly increasing understanding of the regulation and expression of genes and the development of better tools to investigate molecular and cellular mechanisms in model systems ^55^, the hope is that new treatments that can dramatically change the course of illness are on the horizon.
